# Circ_0055625 knockdown inhibits tumorigenesis and improves radiosensitivity by regulating miR-338-3p/MSI1 axis in colon cancer

**DOI:** 10.1186/s12957-021-02234-1

**Published:** 2021-04-21

**Authors:** Chao Gao, Yi Zhang, Yanming Tian, Chun Han, Lan Wang, Boyue Ding, Hua Tian, Chaoxi Zhou, Yingchao Ju, Ale Peng, Qiyao Yu

**Affiliations:** 1 Department of Radiation Oncology, The Fourth Hospital of Hebei Medical University, Shijiazhuang, China; 2 Department of Physiology, Hebei Medical University, Shijiazhuang, 050011 Hebei Province China; 3 Department of Surgery, The Fourth Hospital of Hebei Medical University, Shijiazhuang, China; 4 Department of Experimental Animal Center, The Fourth Hospital of Hebei Medical University, Shijiazhuang, China; 5 Department of Research, The Fourth Hospital of Hebei Medical University, No. 12, Jiankang Road, Shijiazhuang, 050011 Hebei Province China

**Keywords:** Colon cancer, circ_0055625, miR-338-3p, MSI1, Radiosensitivity

## Abstract

**Background:**

Radiotherapy is a main therapeutic method for cancers, including colon cancer. In the current study, we aim to explore the effects of circular RNA (circRNA) circ_0055625 in the progression and radiosensitivity of colon cancer and the underlying mechanism.

**Methods:**

The expression of circ_0055625 and musashi homolog 1 (MSI1) mRNA was detected by quantitative real-time polymerase chain reaction (qRT-PCR). MSI1 protein expression was determined by Western blot. Cell proliferation was assessed by cell counting kit-8 (CCK-8) and colony formation assays. Cell survival fraction, apoptosis, and invasion were investigated by colony formation assay, flow cytometry analysis, and transwell invasion assay, respectively. Cell migration was detected by wound-healing and transwell migration assays. The binding relationship between microRNA-338-3p (miR-338-3p) and circ_0055625 or MSI1 was predicted by online databases and identified by Dual-Luciferase Reporter Assay. The effects of circ_0055625 silencing on the tumor formation and radiosensitivity of colon cancer in vivo were explored by in vivo tumor formation assay.

**Results:**

Circ_0055625 and MSI1 were upregulated in colon cancer tissues and cells relative to control groups. Radiation treatment apparently increased the expression of circ_0055625 and MSI1 in colon cancer cells. Circ_0055625 knockdown or MSI1 silencing repressed cell proliferation, migration, and invasion and promoted cell apoptosis and radiosensitivity in colon cancer. Also, circ_0055625 silencing-mediated effects were attenuated by MSI1 overexpression. Additionally, circ_0055625 silencing reduced MSI1 expression, which could be attenuated by miR-338-3p inhibitor. Mechanically, circ_0055625 acted as a sponge for miR-338-3p to regulate MSI1. Furthermore, circ_0055625 knockdown hindered tumor growth and improved radiosensitivity in vivo.

**Conclusion:**

Circ_0055625 repression inhibited the progression and radioresistance of colon cancer by downregulating MSI1 through sponging miR-338-3p. This result might provide a theoretical basis for improving the therapy of colon cancer with radiation.

## Introduction

Colon cancer is one of the most commonly diagnosed digestive malignancy and poses a heavy burden to human health globally. More than 1,090,000 people are newly diagnosed with colon cancer every year, and about 50% of them die owing to the high rate of metastasis [1]. Clinical data reveal that the combination of operation with radiotherapy can effectively reduce the recurrence rate of colon cancer [2]. Additionally, the therapy for colon cancer can be implemented along NCCN Guidelines. However, radiotherapy increases the proportion of metastatic cells [3]. In term of mechanism, previous data have revealed that irradiation can promote epithelial-mesenchymal transition development by activating the c-jun transcription factor-binding element [4]. More exploration about the mechanism underlying the resistance of colon cancer to radiation is necessary to seek effective therapeutic strategy for colon cancer.

Circular RNA (circRNA), a noncoding RNA, is more stable than linear RNA [5]. As reported, circRNAs are formed by back-splicing events during epithelial mesenchymal transition [6, 7]. Multiple evidences demonstrated that circRNA was involved in the radiosensitivity of cancers. For example, 30 circRNAs were identified to be increased and 37 circRNAs were decreased in lung cancer cells after irradiation (IR) [8]. Liu et al. explained that circ_100367 repressed the radiosensitivity of esophageal squamous cancer cells via binding to microRNA-217 (miR-217) [9]. Li et al. also investigated that circ_000543 silencing improved the radiosensitivity of nasopharyngeal carcinoma cells through targeting miR-9 [10]. Circ_0055625, a cancer-related circRNA, has been found to be upregulated in colon cancer, and promote colon cancer cell growth [11]. But there is few data about the mechanism by which circ_0055625 regulates colon cancer progression and sensitivity to irradiation.

MiRNA, a small RNA with about 20 nucleotides (nts) in size, exerts function through binding to the 3′-untranslated regions (3′UTR) of its target gene [12]. As reported, miR-338-3p was indicated to be an antioncogene in colorectal cancer (CRC) progression. For instance, Liu et al. demonstrated miR-338-3p repression facilitated cell proliferative and migratory capacities, and inhibited cell apoptosis [13]. Zou et al. also uncovered that miR-338-3p was lowly expressed in CRC tissues and cells, and its mimic suppressed CRC progression by targeting metastasis-associated in colon cancer-1 (MACC1) [14]. Additionally, existed studies also confirmed that miR-338-3p overexpression enhanced the sensitivity of colon cancer to irradiation [15]. Musashi 1 (MSI1) is a member of Musashi family and can be regulated by miRNA in cancer progression [16]. Recent data showed that MSI1 was correlated with the progression of cervical cancer [17], lung cancer [18], oral squamous cell carcinoma [19], and colon cancer [20]. In particular, it is found that MSI1 expression is correlated with malignant tumor development and poor prognosis [21, 22]. All these studies indicate that MSI1 functions as a tumor promoter. Our previous data showed that miR-338-3p contained the binding sites of both circ_0055625_and MSI1. Thus, we guessed that circ_0055625 might regulate colon cancer progression and radiosensitivity by miR-338-3p/MSI1 pathway.

In this study, the influences and mechanism of circ_0055625 in the progression of colon cancer and radiosensitivity were unveiled. Whether circ_0055625 silencing regulated colon cancer progression and radiosensitivity by downregulating MSI1 expression through sponging miR-338-3p was revealed. Furthermore, the impacts of circ_0055625 on tumor growth and radiosensitivity were identified in vivo.

## Materials and methods

### Tissue acquirement and storage

Fifty-seven pairs of human colon cancer tissues and adjacent normal tissues (ANT) were obtained from colon cancer patients from the Fourth Hospital of Hebei Medical University. Collected tissues were kept in −80°C ultra-low temperature freezer. The Ethics Committee of The Fourth Hospital of Hebei Medical University agreed with this study. The patients related to this experiment accepted the written informed consents.

### Cell culture

Human colon cancer cell lines (SW620 and SW480) and human normal colorectal mucous membrane cell line FHC were purchased from Otwo Biotech (Shenzhen, China). SW620 and SW480 cells were cultured in Dulbecco’s modified Eagle’s medium (DMEM; HyClone, Logan, UT, USA), and FHC cells were cultivated in Roswell Park Memorial Institute-1640 (RPMI-1640; HyClone) containing 10% fetal bovine serum (FBS; Biosun, Shanghai, China) with 1% streptomycin/penicillin (Gibco, Carlsbad, CA, USA) at 37°C in an incubator with 5% CO_2_.

### Cell transfection

Small interfering RNAs targeting circ_0055625 (si-circ_0055625#1, si-circ_0055625#2 and si-circ_0055625#3) and against MSI1 (si-MSI1#1, si-MSI1#2 and si-MSI1#3), the overexpression plasmid of MSI1 (MSI1), miR-338-3p mimic (miR-338-3p), miR-338-3p inhibitor (anti-miR-338-3p), the small hairpin RNA targeting circ_0055625 (sh-circ_0055625), and control groups (si-NC, vector, miR-NC, anti-NC and sh-NC) were synthesized by GenePharma (Shanghai, China). Cell transfection was conducted using Lipofectamine 3000 (Invitrogen, Carlsbad, CA, USA). The sequences were listed in Table 1.

**Table 1:** Primer sequences and oligonucleotides used in this research

Gene	Sequences of primers/oligonucleotides (from 5′ to 3′)
si-circ_0055625#1	AACAAGGTGGCCCTGTGGAGA
si-circ_0055625#2	AAAACAAGGTGGCCCTGTGGA
si-circ_0055625#3	TTTAAAAAACAAGGTGGCCCT
si-MSI1#1	GGATAAAGTGCTGGCGCAA
si-MSI1#2	GGTTCGGGTTTGTCACGTT
si-MSI1#3	CCAGGTTTCCAAGCCACAA
si-NC	CCATTTACCCGAACGGCAA
miR-338-3p mimic	UCCAGCAUCAGUGAUUUUGUUG
miR-NC	UUUGUACUACACAAAAGUACUG
miR-338-3p inhibitor	CAACAAAAUCACUGAUGCUGGA
Anti-NC	CAGUACUUUUGUGUAGUACAAA
circ_0055625 sense	CTGACATTTAACACTCCCTCC
circ_0055625 anti-sense	GGAACAGGTAGGGCAAGA
MSI1 sense	TACCCAGGTTTCCAAGCCAC
MSI1 anti-sense	CTGTAAGCTCGGGGAGGACT
miR-338-3p sense	CTCACGTCCAGCATCAGTG
miR-338-3p anti-sense	TGGTGTCGTGGAGTCG
GAPDH sense	GGTCACCAGGGCTGCTTT
GAPDH anti-sense	GGAAGATGGTGATGGGATT
U6 sense	CTCGCTTCGGCAGCACA
U6 anti-sense	AACGCTTCACGAATTTGCGT

### Quantitative real-time polymerase chain reaction (qRT-PCR)

Colon cancer tissues and cells were collected and lysed using TransZol (TransGen Biotech, Beijing, China). RNA concentration was measured with NanoDrop-1000 (Thermo Fisher, Waltham, MA, USA) and then reversed transcribed into cDNA using the High-Capacity cDNA kit (Thermo Fisher) or MiX-x™ miRNA synthesis kit (TaKaRa, Dalian, China). For quantifying the levels of circ_0055625, miR-338-3p, and MSI1 mRNA, SuperReal PreMix Color mix (Tiangen, Beijing, China) was performed. Data were assessed with the 2^-∆∆Ct^ method. U6 and glyceraldehyde 3-phosphate dehydrogenase (GAPDH) were selected as references. The sense and antisense primers were displayed in Table 1.

### Western blot analysis

FHC, SW480, and SW620 cells were lysed using RIPA buffer (Beyotime, Jiangsu, China). Lysates were mixed with loading buffer (Thermo Fisher) and boiled in boiling water for 10 min. The samples were loaded on 12% bis-tris-acrylamide gel (Thermo Fisher) and protein bands were transferred onto polyvinylidene fluoride (Millipore, Bradford, MA, USA). After that, the bands were immersed in 5% nonfat milk (Solarbio, Beijing, China) at 4°C for 4 h. The membranes were incubated with anti-MSL1 (1:1000; Abcam, Cambridge, UK) and anti-β-actin (1:10000; Abcam) overnight at 4°C. And the membranes were washed with TBST (Solarbio). The secondary antibody marked with horseradish peroxidase (HRP) (1:5000; Abcam) was used to incubate the membranes at 37°C for 2 h. Protein bands were presented with enhanced chemiluminescence (Millipore). β-actin was employed as a control.

### Cell counting kit-8 (CCK-8) assay

SW480 and SW620 cells were seeded in 96-well plates (5000 cells per well) and cultured for 12 h. si-circ_0055625#3, si-MSI1#2 or MSI1 was transfected into cells with control groups, and cells were cultivated for 24, 48, and 72 h. After that, medium was discarded and 10 μL CCK-8 solution (Beyotime) was used to incubate cells for 4 h. Cell proliferation was determined by detecting the absorbance at 450 nm using microscope reader (Thermo Labsystems, Waltham, MA, USA).

### Colony formation assay

SW480 and SW620 cells were grown in 6-well plates (2×10^5^ cells per well) for 12 h, and si-circ_0055625#3, si-MSI1#2, or MSI1 were transfected into cells with respective controls. Then, the cells were treated with radiation (0, 2, 4, or 6 Gy). Medium was replaced every 3 days. Two weeks later, cell supernatant was discarded, and proliferative colonies were fixed with paraformaldehyde (Sigma, St. Louis, MO, USA) and stained using crystal violet (Sigma). Cell survival fraction or colony-forming ability was analyzing by calculating the number of colonies. A colony was defined when its cell numbers more than 50.

### Flow cytometry analysis

The apoptosis of SW480 and SW620 cells was detected using Annexin V-fluorescein isothiocyanate (Annexin V-FITC)/propidium iodide (PI) detection kit (Solarbio). In short, cells were cultivated in 6-well plates and transfected with plasmids (si-circ_0055625#3, si-MSI1#2, or MSI1) or treated with radiation (6 Gy). Trypsin (Thermo Fisher) was performed to digest cells, and cells were then washed with phosphate buffer solution (PBS; Solarbio). Following that, cells were mixed with binding buffer (Solarbio) and centrifuged at 260 rpm for 10 min. After that, binding buffer was used to suspend the cells, and Annexin V-FITC (Solarbio) and PI (Solarbio) were conducted to incubate cells in dark. Results were demonstrated with flow cytometry (BD Biosciences, San Diego, CA, USA).

### Wound-healing assay

The migration of SW480 and SW620 cells was determined by wound-healing assay. Shortly, cells were seeded in 6-well plates and cultured for 16 h. And cells were treated with si-circ_0055625#3, si-MSI1#2, MSI1, or radiation (6 Gy). Wounds were made with a 10-μL pipette tip when the confluence of SW480 and SW620 cells reached 100%. Then, PBS (Solarbio) was used to wash cells and FBS-free DMEM (HyClone) was added into the plates. 24 h later, cell migration was determined by measuring the areas occupied by migrated cells under microscope (Olympus, Tokyo, Japan) with ×100 magnification.

### Transwell migration and invasion assays

The migration and invasion of SW480 and SW620 cells were revealed by Transwell chambers without or with Matrigel (Corning, New York, Madison, USA), respectively. In short, cells were cultured in 24-well plates at a density of 5×10^4^ cells each well and treated with si-circ_0055625#3, si-MSI1#2, MSI1, or radiation (6 Gy). And serum-free DMEM (HyClone) was added into the upper chambers, and DMEM contained 15% FBS (Biosun) was placed into the lower chambers. 24 h later, the chambers were removed from the plates. Then, the cells were washed with PBS (Solarbio). Thereafter, methanol (Beyotime) and crystal violet (Beyotime) were performed to immobilize and dye cells, respectively. Results were unveiled via microscope (Olympus) with ×100 magnification.

### Dual-Luciferase Reporter Assay

The binding sequence between miR-338-3p and circ_0055625 or MSI1 3′UTR was predicted by Starbase online database. The wild-type (WT) sequences of both circ_0055625 and MSI1 3′UTR bound by miR-338-3p were fused into pmirGLO vector (Promega, Madison, WI, USA) and named as WT-circ_0055625 and WT-MSI1 3′UTR. The sequences of both circ_0055625 and MSI1 3′UTR targeted via miR-338-3p were mutated, and mutant (MUT) circ_0055625 and MSI1 3′UTR were cloned into pmirGLO vector (Promega), which were named as MUT-circ_0055625 and MUT-MSI1 3′UTR. Conducted plasmids were transfected into SW480 and SW620 cells with miR-338-3p mimic or miR-NC using DharmaFECT 4 (Thermo Fisher). Luciferase activity was detected with Dual-Lucy Assay Kit (Solarbio) with *Ranilla* Luciferase activity as a control.

### In vivo tumor formation assay

Charles River (Beijing, China) provided BALB/c nude mice (5 weeks old). All mice were fed in pathogen-free environment. Nude mice were divided into the 4 groups (sh-NC group, sh-circ_0055625 group, sh-NC+radiation group, and sh-circ_0055625+radiation group, *N*=6 per group). SW480 cells transfected with sh-circ_0055625 or sh-NC were suspended in PBS (6×10^6^/mL), and 200 μL cell suspension was injected into the right flank of mice. And tumor volume was measured every 3 days. Cells in radiation group and sh-circ_0055625+radiation group were treated with radiation (6 Gy per day) for 3 days on the 12th day after injection. 27 days, mice were euthanized and tumors were excised. The volume and weight of tumors were measured. A part of every tumor was kept at −80°C in freezer for assessing the knockdown efficiency of sh-circ_0055625. The Animal Care and Use Committee of the Fourth Hospital of Hebei Medical University agreed with this study.

### Data analysis

All data were gotten based on 3 replicates and analyzed using SPSS 21.0 software (IBM, Somers, NY, USA). The linear relationship between circ_0055625 and MSI1 was disclosed by Spearman correlation analysis. Data were shown as means ± standard deviations (SD) and assessed with two-tailed Student’s *t* tests, Wilcoxon rank-sum test, or one-way analysis of variance (ANOVA). *P* value < 0.05 was considered statistically significant.

## Results

### Circ_0055625 and MSI1 were highly expressed in colon cancer tissues and cells with poor survival rate

**Fig. 1:**
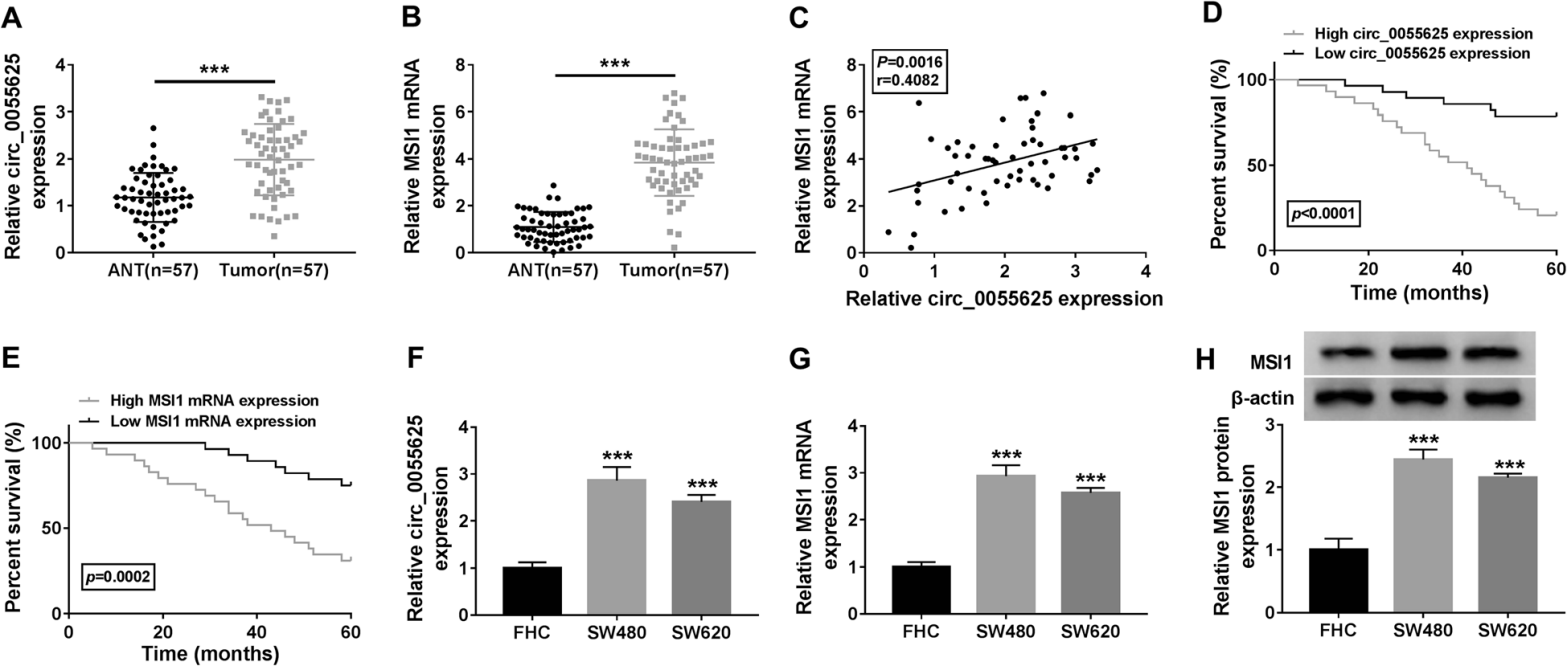
Circ_0055625 and MSI1 expression were upregulated in colon cancer tissues and cells with poor survival rate. **a**, **f** Circ_0055625 expression was determined by qRT-PCR in colon cancer tissues, normal colon tissues, and FHC, SW480, and SW620 cells. **b**, **g**, and **h** The mRNA and protein expression of MSI1 were detected by qRT-PCR and Western blot, respectively, in colon cancer tissues, normal colon tissues, and FHC, SW480, and SW620 cells. **c** Spearman correlation analysis was performed to reveal the linear relationship between circ_0055625 expression and MSI1 expression in colon cancer tissues. **d**, **e** Kaplan-Meier method was conducted to analyze the relationship between the survival rate of colon cancer patients and circ_0055625 or MSI1 expression. **P<0.01, ***P<0.001, and ****P<0.0001

In order to determine the properties of circ_0055625 and MSI1 in the progression and radiosensitivity of colon cancer, their expression was firstly detected in colon cancer tissues and cells. Results showed that circ_0055625 expression was upregulated (Fig. 1a, f), and MSI1 expression was also visibly increased (Fig. 1b, g, and h) in colony cancer tissues as well as SW480 and SW620 cells compared with normal tissues and FHC cells. In addition, we found that circ_0055625 expression was positively related to MSI1 expression (Fig. 1c). Furthermore, Kaplan-Meier methods demonstrated that both high circ_0055625 and MSI1 expression were correlated with poor survival rate of colon cancer patients (Fig. 1d, e). These data indicated that circ_0055625 and MSI1 might play vital roles in colon cancer progression.

### Circ_0055625 and MSI1 expression were upregulated in response to IR in colon cancer cells

**Fig. 2:**
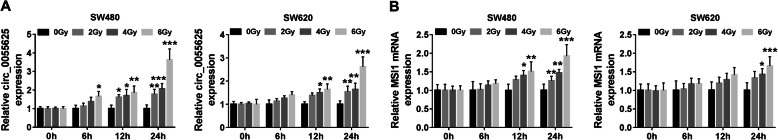
Radiation treatment increased the expression of both circ_0055625 and MSI1 in colon cancer cells. **a**, **b** The expression of circ_0055625 and MSI1 was detected by qRT-PCR at the indicated time points (0, 6, 12, and 24 h) after radiation treatment (0, 2, 4, and 6 Gy) in SW480 and SW620 cells. *P<0.05, **P<0.01, and ***P<0.001

The effects of radiation on the expression of circ_0055625 and MSI1 were further studied. Results demonstrated that radiation treatment (4 or 6 Gy) apparently increased circ_0055625 expression under IR treatment in SW480 and SW620 cells (Fig. 2a). Also, MSI1 expression was significantly increased in SW480 and SW620 cells treated with radiation (4 or 6 Gy) (Fig. 2b). These results suggested that circ_0055625 and MSI1 might regulate the outcome of radiation for colon cancer.

### Circ_0055625 knockdown repressed cell proliferation, migration, and invasion, whereas promoted cell apoptosis and radiosensitivity in colon cancer

**Fig. 3:**
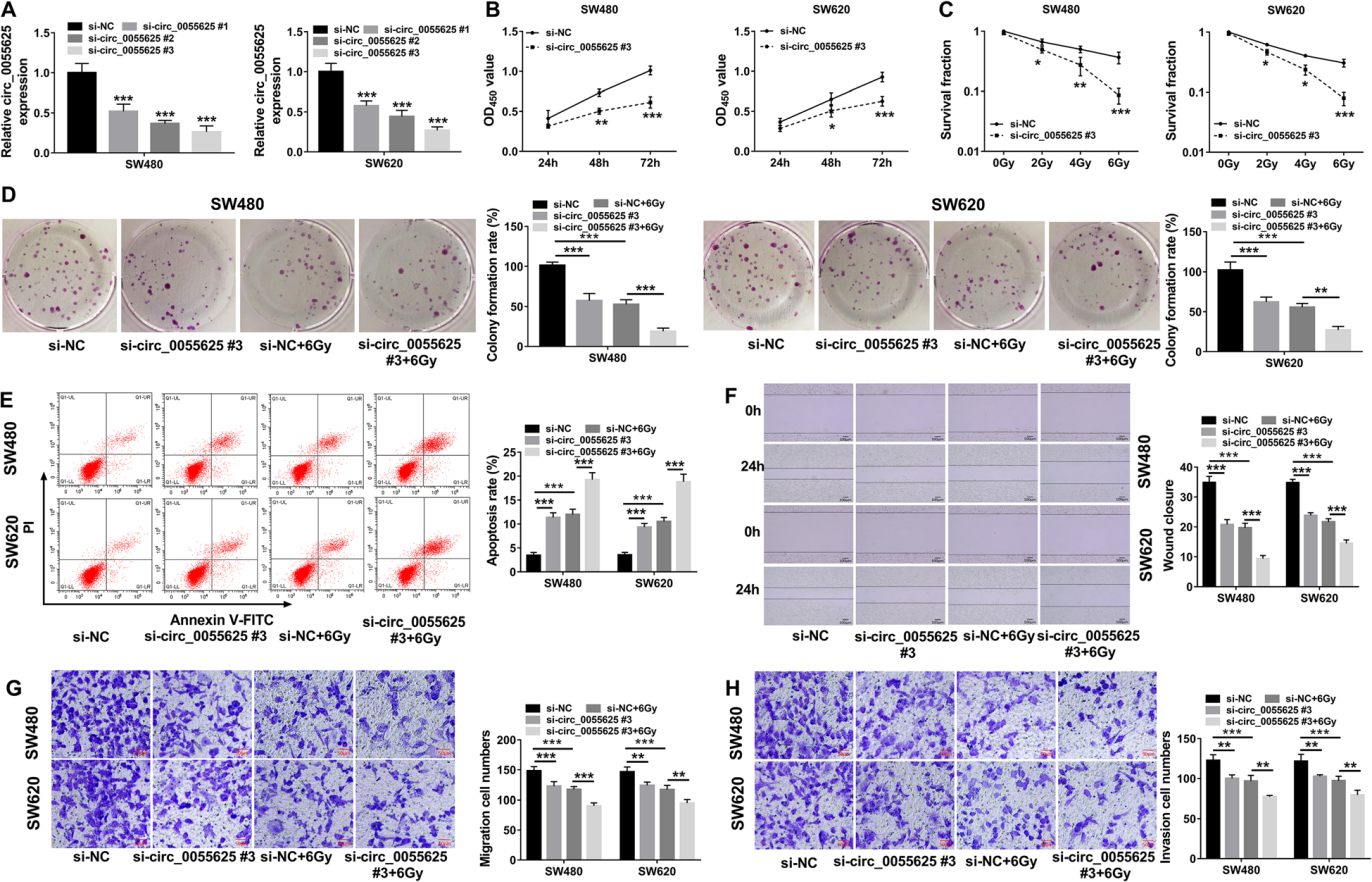
Circ_0055625 knockdown inhibited tumor progression and enhanced radiosensitivity in colon cancer. **a** The knockdown efficiency of si-circ_0055625#1, si-circ_0055625#2, or si-circ_0055625#3 was detected by qRT-PCR in SW480 and SW620 cells. **b** CCK-8 assay was performed to determine the effect of circ_0055625 silencing on the proliferation of SW480 and SW620 cells. **c** The impacts of circ_0055625 absence on the survival fraction of SW480 and SW620 cells after treatment of radiation (0, 2, 4, and 6 Gy) were investigated by colony formation assay. **d** The effects of circ_0055625 silencing on cell colony-forming ability with or without radiation treatment were investigated by colony formation assay in SW480 and SW620 cells. **e** Flow cytometry analysis was employed to elucidate the impacts of circ_0055625 depletion on cell apoptosis with or without radiation treatment in SW480 and SW620 cells. **f**, **g** Wound-healing and transwell migration assays were carried out to unveil the impacts of circ_0055625 repression on cell migration under radiation presence or absence in SW480 and SW620 cells. **h** Transwell invasion assay were carried out to unveil the impacts of circ_0055625 repression on cell invasion under radiation presence or absence in SW480 and SW620 cells. *P<0.05, **P<0.01, and ***P<0.001

To further disclose the impacts of circ_0055625 on the radiosensitivity and development of colon cancer, the interfering plasmids of circ_0055625 were built and respective knockdown efficiency was identified. QRT-PCR results displayed that si-circ_0055625#1, si-circ_0055625#2, or si-circ_0055625#3 obviously downregulated circ_0055625 expression (Fig. 3a), and si-circ_0055625#3 was chosen for subsequent study because of its most effective impact in reducing circ_0055626. Subsequently, results presented that circ_0055625 knockdown repressed the proliferation of SW480 and SW620 cells (Fig. 3b). Colony formation assay showed that circ_0055625 knockdown repressed cell colony-forming ability and facilitated the inhibitory effect of radiation on cell colony-forming ability (Fig. 3d). In addition, circ_0055625 silencing reduced the survival fraction of SW480 and SW620 cells under radiation treatment in a dose-dependent manner (Fig. 3c). Furthermore, circ_0055625 knockdown suppressed cell migration and invasion and enhanced the repressive effects of radiation on cell migration and invasion in SW480 and SW620 cells (Fig. 3f, g, and h). The above data demonstrated that circ_0055625 knockdown repressed the progression of colon cancer and potentiated the effects of radiation.

### MSI1 silencing repressed the progression and radioresistance of colon cancer

**Fig. 4:**
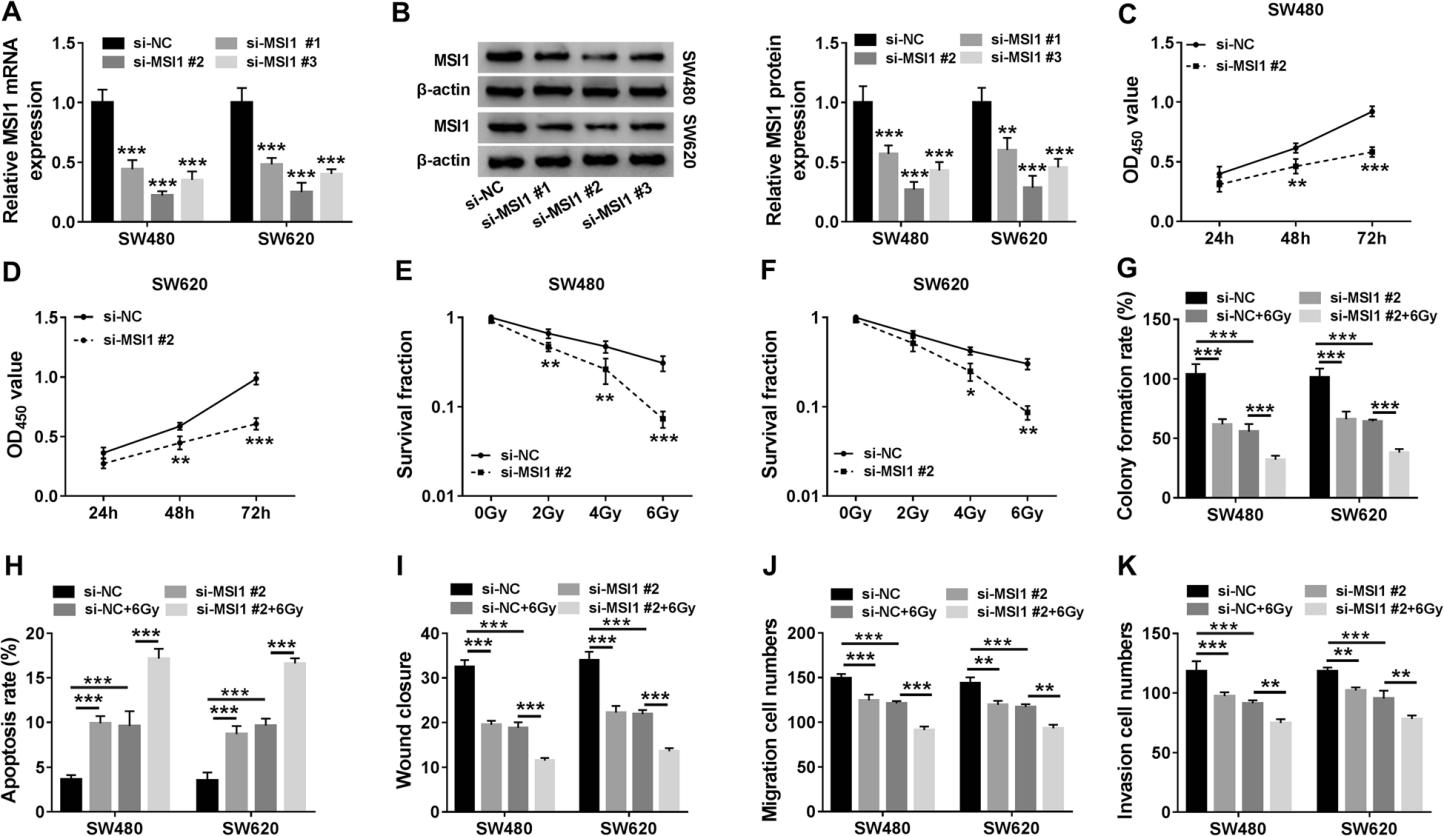
MSI1 knockdown inhibited tumorigenesis and radioresistance in colon cancer. **a**, **b** The mRNA and protein expression of MSI1 were severally detected by qRT-PCR and Western blot after si-MSI1#1, si-MSI1#2, or si-MSI1#3 transfection in SW480 and SW620 cells. **c**, **d** The effect of MSI1 silencing on the proliferation of SW480 and SW620 cells was revealed by CCK-8 assay. **e**, **f** Colony formation assay was conducted to study the effect of MSI1 repression on the survival fraction of SW480 and SW620 cells after treatment of radiation (0, 2, 4, and 6 Gy). **g** The effects of MSI1 knockdown on cell colony-forming ability with radiation presence or absence were elucidated by colony formation assay in SW480 and SW620 cells. **h** The impacts of MSI1 depletion on cell apoptosis with radiation presence or absence were presented by flow cytometry analysis in SW480 and SW620 cells. **i**, **j** Wound-healing and Transwell migration assays were performed to determine the impacts of MSI1 knockdown on the migration of SW480 and SW620 cells with radiation presence or absence. **k** Transwell invasion assay was performed to determine the impacts of MSI1 knockdown on the invasion of SW480 and SW620 cells with radiation presence or absence. *P<0.05, **P<0.01, and ***P<0.001

The effects of MSI1 on colon cancer progression and radioresistance were revealed in this part. Our results primarily showed that the mRNA and protein expression of MSI1 were dramatically downregulated by si-MSI1#1, si-MSI1#2, or si-MSI1#3 (Fig. 4a, b), and si-MSI1#2 was employed for further study as it has the most effective impact in downregulating MSI1 expression. Subsequently, CCK-8 assay presented that MSI1 knockdown repressed cell proliferation in SW480 and SW620 cells (Fig. 4c, d). Colony formation assay also showed that MSI1 silencing decreased the survival fraction of SW480 and SW620 cells under radiation in a dose-dependent manner (Fig. 4e, f). In addition, MSI1 silencing repressed cell colony-forming ability and enhanced the inhibitory effect of radiation on cell colony-forming ability (Fig. 4g). Flow cytometry analysis revealed that MSI1 repression facilitated cell apoptosis and the impact of radiation treatment on cell apoptosis (Fig. 4h). Furthermore, MSI1 was disclosed to inhibit cell migration and invasion and accelerate the repressive impacts of radiation exposure on cell migration and invasion (Fig. 4i–k). Hence, these evidences indicated that MSI1 knockdown inhibited cell proliferation, migration, and invasion and promoted cell apoptosis and radiosensitivity in colon cancer in vitro.

### MSI1 overexpression attenuated the inhibitory effects of circ_0055625 silencing on the progression and radioresistance of colon cancer

**Fig. 5:**
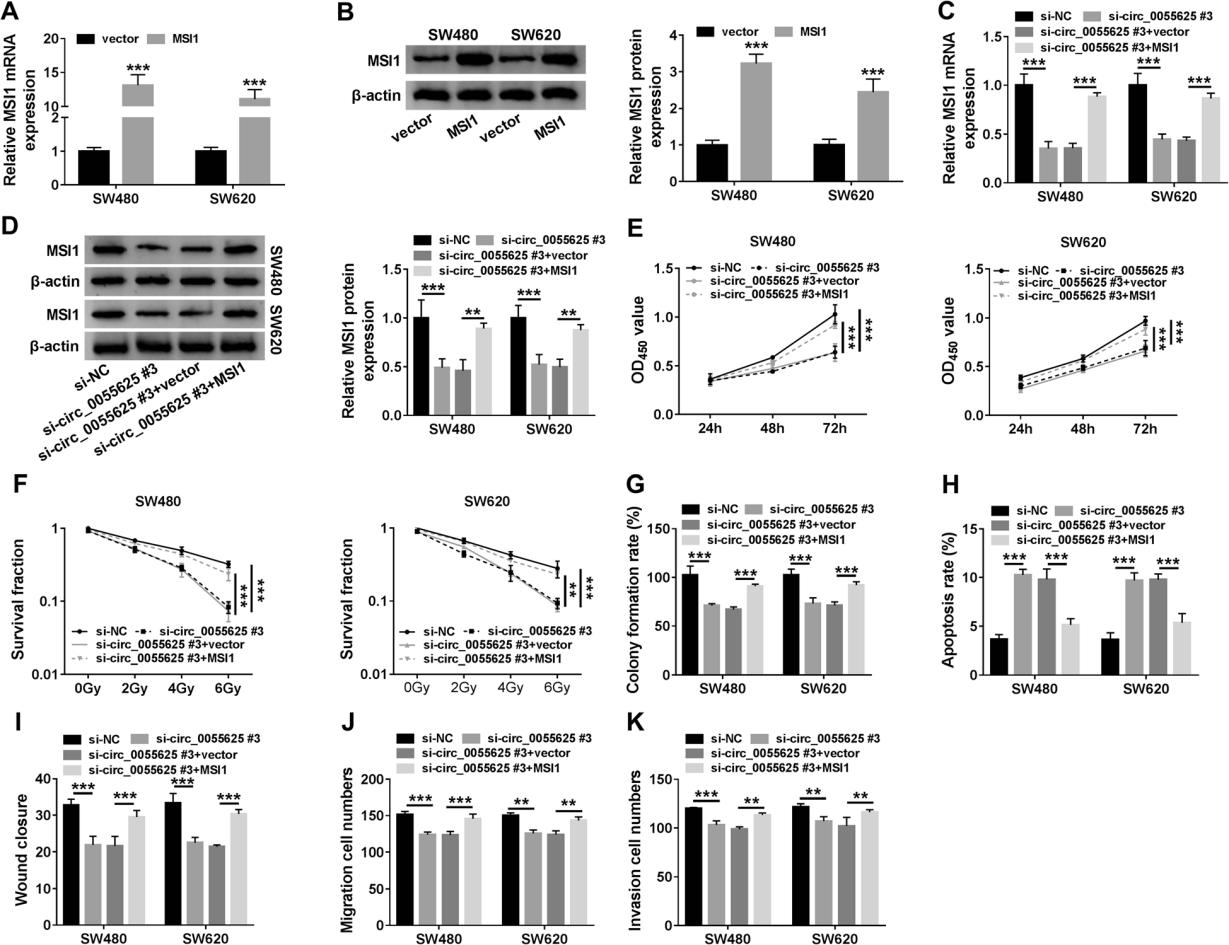
MSI1 overexpression impaired the inhibitory impacts of circ_0055625 knockdown on the progression and radioresistance of colon cancer. **a**, **b** The mRNA and protein expression of MSI1 were detected by qRT-PCR and Western blot, respectively, in SW480 and SW620 cells transfected with MSI1 or vector. **c**, **d** The effects between circ_0055625 silencing and MSI1 overexpression on the mRNA and protein expression of MSI1 were determined by qRT-PCR and Western blot, respectively, in SW480 and SW620 cells. **e**, **g** CCK-8 and colony formation assays were performed to disclose the effects between circ_0055625 silencing and enforced MSI1 expression on the proliferation of SW480 and SW620 cells. **f** The effects between circ_0055625 depletion and enforced MSI1 expression on the survival fraction of SW480 and SW620 cells under radiation treatment (0, 2, 4, and 6 Gy) were demonstrated by colony formation assay. **h** The influences between circ_0055625 absence and ectopic MSI1 expression on the apoptosis of SW480 and SW620 cells were unveiled by flow cytometry analysis. **i**, **j** Wound-healing and transwell migration assays were carried out to present the impacts between circ_0055625 silencing and MSI1 overexpression on the migration of SW480 and SW620 cells. **k** Transwell invasion assay was performed to present the impacts between circ_0055625 silencing and MSI1 overexpression on the invasion of SW480 and SW620 cells. **P<0.01 and ***P<0.001

Given the effects of both circ_0055625 and MSI1 on the progression and radiosensitivity of colon cancer, the relationship of circ_0055626 and MSI1 in mediating the progression and radioresistance of colon cancer was further explored. Data initially showed that the mRNA and protein expression of MSI1 were dramatically upregulated in SW480 and SW620 cells transfected with MSI1 (Fig. 5a, b), suggesting the high efficiency of MSI1 in upregulating MSI1 expression. Subsequently, it was found that circ_0055625 knockdown inhibited the mRNA and protein expression of MSI1, whereas these effects were restored by MSI1 overexpression (Fig. 5c, d). Circ_0055625 knockdown also suppressed the proliferation of SW480 and SW620 cells; however, MSI1 overexpression attenuated this impact (Fig. 5e, g). Similarly, circ_0055625 knockdown reduced the survival fraction of SW480 and SW620 cells in a dose-dependent fashion after radiation treatment, but the effect was restrained by MSI1 overexpression (Fig. 5f). And the apoptosis of SW480 and SW620 cells was induced by circ_0055625 repression, whereas enforced MSI1 expression hindered this effect (Fig. 5h). Furthermore, ectopic MSI1 expression impaired the inhibitory influences of circ_0055625 silencing on the migration and invasion of SW480 and SW620 cells (Fig. 5i–k). These findings suggested that circ_0055625 could regulate the progression and radiosensitivity of colon cancer by MSI1.

### Circ_0055625-induced MSI1 expression by acting as a sponge of miR-338-3p

**Fig. 6:**
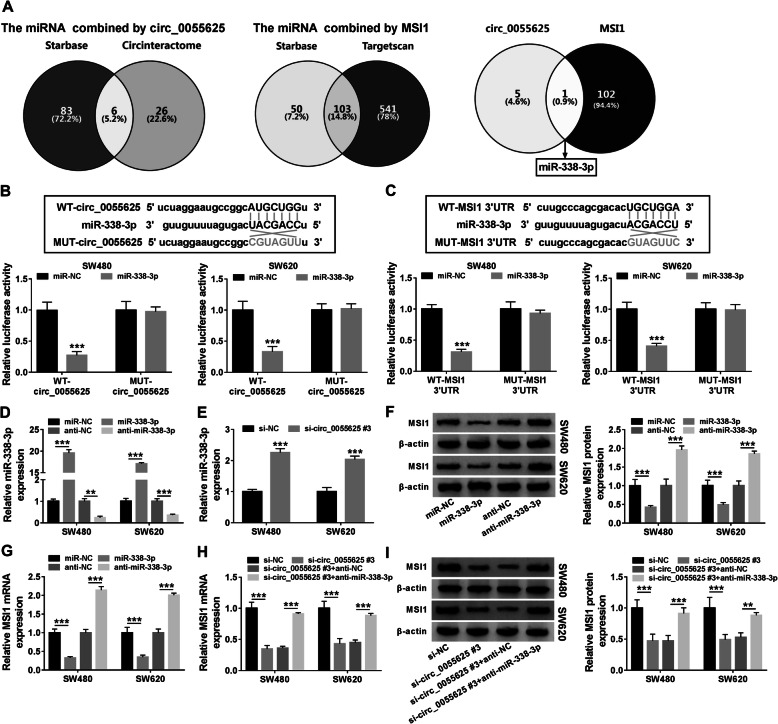
Circ_0055625 regulated MSI1 expression by binding to miR-338-3p. **a** The miRNAs associated with circ_0055625 and MSI1 were predicated by Starbase, Circinteractome, or Targetscan online database. **b**, **c** The binding sequences between miR-338-3p and circ_0055625 or MSI1 were predicted by Starbase online database and identified by Dual-Luciferase Reporter Assay. **d** The efficiency of miR-338-3p mimic and inhibitor in upregulating or downregulating miR-338-3p expression was detected by qRT-PCR. **e** The effect of circ_0055625 knockdown on miR-338-3p expression was investigated by qRT-PCR in SW480 and SW620 cells. **f**, **g** The impacts of miR-338-3p mimic or inhibitor on the mRNA and protein expression of MSI1 were determined by qRT-PCR and Western blot, respectively, in SW480 and SW620 cells. **h**, **i** The influences between circ_0055625 repression and miR-338-3p inhibitor on the mRNA and protein levels of MSI1 were determined by qRT-PCR and western blot, respectively, in SW480 and SW620 cells. **P<0.01 and ***P<0.001

The study continued to unveil the mechanism underlying the reversed effect of MSI1 on circ_0055625 silencing-mediated action. Considering that circRNA usually acted as a competing endogenous RNA (ceRNA) to weaken the activity of miRNA [23], we tried to screen the miRNAs associated with both circ_0055625 and MSI1. As we expected, there were 6 miRNAs containing the binding sites of circ_0055625, and 103 miRNAs containing the binding sequence of MSI1. Among these miRNAs, only miR-338-3p was simultaneously associated with both circ_0055625 and MSI1 (Fig. 6a). Thus, miR-338-3p was employed as a candidate. The binding sites between miR-338-3p and circ_0055625 or MSI1 were shown in Fig. 6b, c. Subsequently, Dual-Luciferase Reporter Assay presented that the luciferase activity of WT-circ_0055625 and miR-338-3p mimic group was dramatically repressed, whereas the luciferase activity had no obvious change in MUT-circ_0055625 and miR-338-3p mimic co-transfection group (Fig. 6b). Results also exhibited that luciferase activity was apparently inhibited in WT-MSI1 3′UTR and miR-338-3p mimic group, but not in MUT-MSI1 3′UTR and miR-338-3p mimic group (Fig. 6c). In order to disclose the effects of miR-338-3p mimic or inhibitor on MSI1 expression, the efficiency of miR-338-3p mimic and inhibitor in increasing or decreasing miR-338-3p was detected. Results showed that miR-338-3p mimic significantly upregulated miR-338-3p expression and miR-338-3p inhibitor obviously downregulated miR-338-3p expression (Fig. 6d), proving the high efficiency of miR-338-3p mimic and inhibitor in increasing or decreasing miR-338-3p. QRT-PCR results showed that circ_0055625 knockdown notably increased miR-338-3p expression (Fig. 6e). In addition, the mRNA and protein expression of MSI1 were effectively decreased by miR-338-3p mimic and upregulated by miR-338-3p inhibitor (Fig. 6f, g). Data also showed that circ_0055625 silencing dramatically downregulated the mRNA and protein expression of MSI1, whereas these effects were attenuated after miR-338-3p depletion (Fig. 6h, i). These results indicated that circ_0055625 regulated MSI1 expression by sponging miR-338-3p.

### Circ_0055625 knockdown repressed tumor formation and enhanced radiosensitivity in colon cancer in vivo

**Fig. 7:**
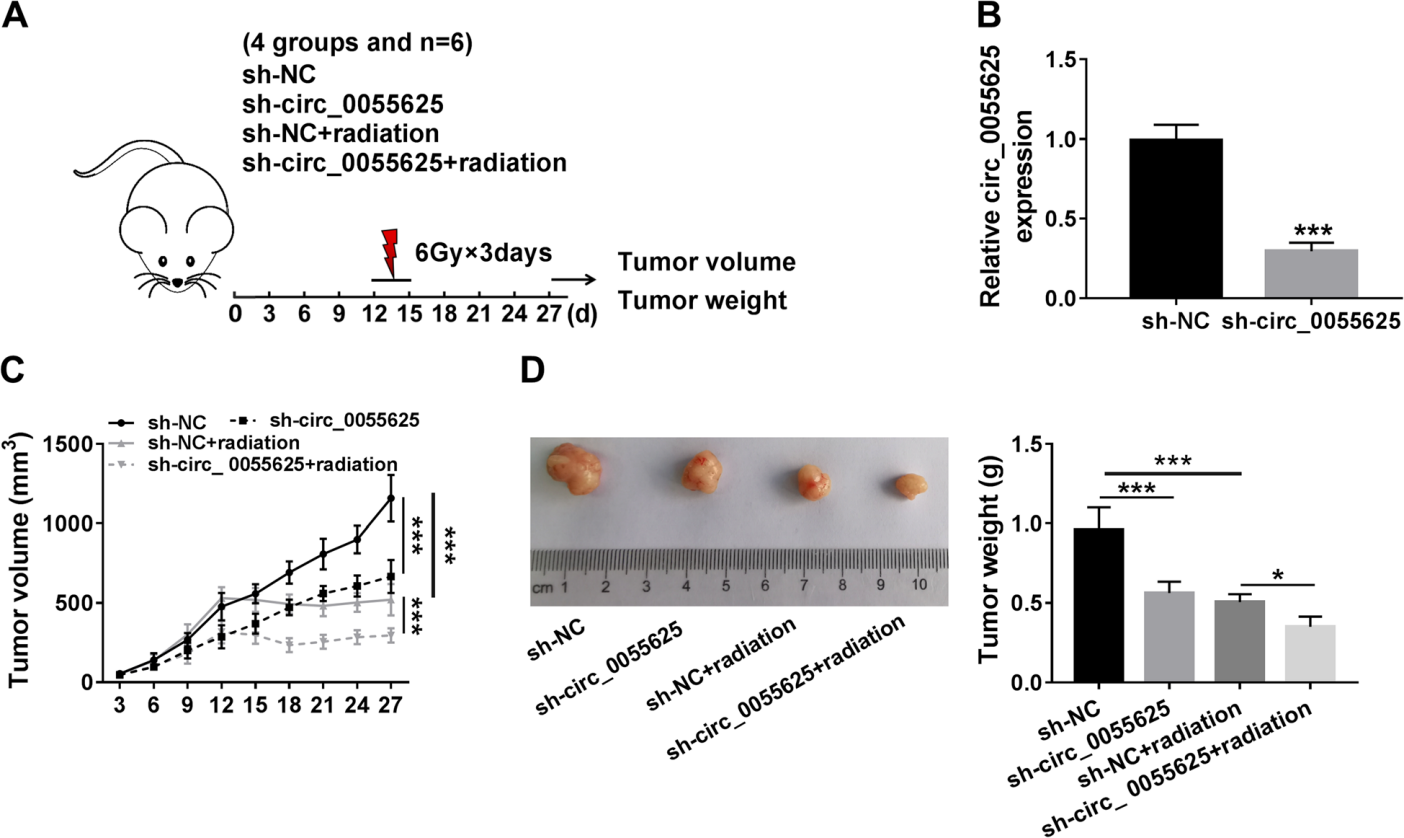
Circ_0055625 silencing inhibited the tumorigenesis and radioresistance of colon cancer cells in vivo. **a** The schematic diagram presented the procedure of in vivo tumor formation. **b** The silencing efficiency of sh-circ_0055625 was determined by qRT-PCR in vivo. **c**, **d** The effects of circ_0055625 knockdown on the radiosensitivity as well as tumor volume and weight in vivo were determined. *P<0.05 and ***P<0.001

In order to further demonstrate the effects of circ_0055625 on the growth and radiosensitivity of colon cancer, in vivo tumor formation assay was employed. As shown in Fig. 7a, mice were divided into 4 groups and treated with radiation (6Gy each day) on the 12th day for 3 days after injection. Results firstly showed that circ_0055625 expression was dramatically downregulated after sh-circ_0055625 transfection (Fig. 7b), suggesting that the interfering plasmid of circ_0055625 was successfully built. Subsequently, data showed that circ_0055625 silencing suppressed tumor volume and weight, and circ_0055625 knockdown facilitated the inhibitory effects of radiation on tumor volume and weight (Fig. 7c, d). These data showed that circ_0055625 silencing suppressed tumor growth and contributed to radiosensitivity in vivo.

## Discussion

Accumulating evidences suggest that circRNA can modulate cancer sensitivity to IR [24, 25]. In colon cancer, Zhang et al. explained that circ_0055625 expression was apparently increased in colon cancer tissues and was correlated with tumor metastasis [11]. However, the studies on the mechanism of colon cancer progression and radiosensitivity regulated by circ_0055625 have not been fully addressed. In this study, we provided evidences that circ_0055625 mediated colon cancer development and sensitivity to IR by miR-338-3p/MSI1 pathway.

Previous data showed that the radiosensitivity of cancers could be regulated by circRNAs, such as circ_0086720 [26], circRNA versican [27], and circRNA Cyclin B2 [28]. Another circRNA, circ_0055625, has been revealed to mediate the resistance of colon cancer to leucovorin [29]. However, there was few data about circ_0055625 regulating the sensitivity of colon cancer to radiation. In the present research, the role of circ_0055625 in modulating radiosensitivity was revealed for the first time. Our findings presented that circ_0055625 expression was visibly upregulated in colon cancer tissues, and that circ_0055625 knockdown suppressed cell proliferation as well as migratory and invasive abilities, which was proved by the study of Zhang et al. [11]. Additionally, we found that radiation treatment increased circ_0055625 level in colon cancer cells and that circ_0055625 knockdown accelerated cell apoptosis and sensitivity to IR in colon cancer. Furthermore, data presented that circ_0055625 knockdown reduced the volume and weight of the forming tumors and improved the inhibitory impact of radiation on tumor growth. Our evidences explained that circ_0055625 was a cancer promoter in colon cancer progression and improved colon cancer sensitivity to radiation.

Existed study has investigated that (-)-gossypol suppresses colon cancer process via modulating MSI1 [30]. Li et al. corroborated that MSI1 repression hindered cell proliferation, migration, and invasion in colon cancer [31]. Additionally, it was revealed that MSI1 absence facilitated cell apoptosis in colon adenocarcinoma [32]. These evidences displayed the promoting role of MSI1 in colon cancer progression. In this research, MSI1 expression was apparently upregulated in colon cancer tissues and cells and increased in colon cancer cells treated with radiation. In addition, results also showed that MSI1 repression restrained cell proliferation, migration, and invasion, whereas improved cell apoptosis in colon cancer. Currently, it has been reported that MSI1 depletion enhances the sensitivity of glioblastoma multiforme to radiation through weakening tumor invasion [33]. In line with the above finding, we also demonstrated the promoting role of MSI1 silencing in radiation sensitivity. Furthermore, MSI1 overexpression also attenuated the impact of circ_0055625 absence on tumor development and radiosensitivity in colon cancer. These evidences indicated that circ_0055625 knockdown suppressed the development of colon cancer and improved sensitivity of colon cancer to IR by regulating MSI1. CircRNA commonly acts as a ceRNA to repress the activity of miRNA, which further leads to the downregulation of the miRNA expression as well as the decrease of the mRNA targeted by miRNA [34]. Based on the connection, we wondered whether there was the miRNA that acted as a target gene of circ_0055625 and bound to MSI1.

The progression of colon cancers involved lots of miRNAs, such as miR-141 [35], miR-219a-1 [36], and miR-503-5p [37]. MiR-338-3p, a miRNA, was found to play vital parts in colon cancer cell processes. For example, Zou et al. presented that miR-338-3p was lowly expressed and inhibited cell proliferation in CRC [14]. Wang et al. also explained circ_0001313 absence facilitated colon cancer sensitivity to radiation, and inhibited cell proliferation through binding to miR-338-3p [15]. These finding suggested miR-338-3p acted as a tumor suppressor and could promote colon cancer radiosensitivity. Interestingly, our data exhibited that circ_0055625 was associated with miR-338-3p and miR-338-3p bound to MSI1. Furthermore, results explained that circ_0055625 depletion downregulated MSI1 expression through binding to miR-338-3p. Importantly, the anti-cancer role of miR-338-3p was also found in cervical cancer [38], gastric cancer [39], prostate cancer [40], and hypopharyngeal carcinoma [41], providing the possible of miR-338-3p inhibitor as an anti-cancer drug.

The disadvantage of this study is the lack of the data about the effects of colon cancer process and radiosensitivity regulated by miR-338-3p, which will be studied in further study. In addition, the study of mouse model assay about the mechanism is incomplete in the present paper, which will be demonstrated in the future.

Summary, the expression of circ_0055625 and MSI1 was increased in colon cancer tissues and cells, and radiation treatment increased circ_0055625 and MSI1 expression. Either circ_0055625 knockdown or MSI1 silencing hindered cell proliferation, migration, and invasion, whereas elevated cell apoptosis and radiosensitivity in colon cancer cells. In addition, circ_0055625 was associated with miR-338-3p and miR-338-3p bound to MSI1. Furthermore, circ_0055625 knockdown hindered tumor formation and improved radiosensitivity in vivo. Our finding provides a new mechanism for studying noncoding RNAs therapy of colon cancer and also implicates that circ_0055625 and MSI1 have potential to serve as biomarkers for colon cancer radiotherapy. Also, circ_0055625/MSI1-knockdown-directed biomarkers may be employed in further developing targeted drugs of colon cancer.

## Data Availability

Not applicable.

## Abbreviations

MSI1: Musashi homolog 1
CRC: Colorectal cancer
ANT: Adjacent normal tissues
SD: Standard deviations
ANOVA: Analysis of variance
