# Correction: Circ_0055625 knockdown inhibits tumorigenesis and improves radiosensitivity by regulating miR-338-3p/MSI1 axis in colon cancer

**DOI:** 10.1186/s12957-024-03391-9

**Published:** 2024-04-26

**Authors:** Chao Gao, Yi Zhang, Yanming Tian, Chun Han, Lan Wang, Boyue Ding, Hua Tian, Chaoxi Zhou, Yingchao Ju, Ale Peng, Qiyao Yu

**Affiliations:** 1 Department of Radiation Oncology, The Fourth Hospital of Hebei Medical University, Shijiazhuang, China; 2 Department of Physiology, Hebei Medical University, Shijiazhuang, 050011 Hebei Province China; 3 Department of Surgery, The Fourth Hospital of Hebei Medical University, Shijiazhuang, China; 4 Department of Experimental Animal Center, The Fourth Hospital of Hebei Medical University, Shijiazhuang, China; 5 Department of Research, The Fourth Hospital of Hebei Medical University, No. 12, Jiankang Road, Shijiazhuang, 050011 Hebei Province China

**Correction: World Journal of Surgical Oncology (2021) 19:131.**

10.1186/s12957-021-02234-1.

Following the publication of the original article [1], the author reported that they miss to include the following Funding statement:

Funding.

This research was supported by the Natural Science Foundation of Hebei Province (H2020206311) and the Central Guiding Local Science and Technology Development Fund Project (Hebei Science and Technology Department Project) (226Z7712G).

The original article has been updated.

## References

[1] Gao C, Zhang Y, Tian Y (2021). Circ_0055625 knockdown inhibits tumorigenesis and improves radiosensitivity by regulating miR-338-3p/MSI1 axis in colon cancer. World J Surg Onc.

